# U-Shaped Optical Fiber Probes Coated with Electrically Doped GQDs for Humidity Measurements

**DOI:** 10.3390/polym13162696

**Published:** 2021-08-12

**Authors:** Hsin-Yi Wen, Hsiang-Cheng Hsu, Yao-Tung Tsai, Wen-Kai Feng, Chih-Lang Lin, Chia-Chin Chiang

**Affiliations:** 1 Department of Green Energy and Environmental Resources, Chang Jung Christian University, Tainan City 71101, Taiwan; hywen@mail.cjcu.edu.tw; 2 Department of Mechanical Engineering, National Kaohsiung University of Science Technology, Kaohsiung 807618, Taiwan; gn1204774@gmail.com (H.-C.H.); ydung100@gmail.com (Y.-T.T.); F109142146@nkust.edu.tw (W.-K.F.); 3 Department of Aviation Management, Chinese Air Force Academy, Kaohsiung 82047, Taiwan; 4 Department Institute of Biomedical Engineering and Materials Science, Central Taiwan University of Science and Technology, Taichung 40601, Taiwan; cllin101943@ctust.edu.tw

**Keywords:** optical fiber probes, U shape, electrospinning, graphene quantum dots

## Abstract

The influence of the bending radius on the sensitivity of the graphene quantum dots (GQDs)-coated probe is experimentally investigated for a U-shaped probe. The fiber is bent into a U shape using the optic fiber flame heating method, and the optic fiber is enclosed in a glass tube to increase the stability of the probe. The surface of the U-shaped optical fiber was coated with electrospun fibers formed via electrospinning. Polymer materials doped with GQDs are applied to U-shaped optical fiber as humidity sensors. Graphene quantum dot nanofibers on the U-shaped optical fiber sensor to form a network structure of graphene quantum dots U-shape fiber sensor (GQDUS). The polymer network structure absorbs water molecules, which in turn affects the bending radius of the optical fiber, and changes the optical fiber spectrum. Graphene quantum dots provide optical enhancement benefits, which in turn increase the sensitivity of fiber optic sensors. The spectra monitoring system consists of an optical spectrum analyzer (OSA) and an amplified spontaneous emission (ASE). This system can be used to detect humidity changes between 20% RH and 80% RH in the chamber. Our results indicate promising applications for quantum dots probe sensors from electrospun nanofibers increasing sensitive environmental monitoring. As such, it could be of substantial value in optical sensors detection.

## 1. Introduction

Optical fibers are part of everyday life, especially in communications, where they are used to convey information. Optical fibers have various advantages: low weight, small radius, high stability, high sensitivity, imperviousness to electromagnetic interference, and a high tolerance for harsh environmental conditions [1,2]. Due to their high sensitivities, optical fibers are widely applied in communications and have also displayed excellent performance in sensing and measurement applications [3]. Numerous researchers have modified optical fibers to work as sensors and improved their sensitivity and accuracy when measuring physical quantities. Advances in optical fiber technology have increased the range and utility of optical fiber applications [4,5]. Researchers have studied the uses of optical fibers in medicine, defense, and aerospace. A wide range of optical fiber sensors has been developed based on their response to various environmental factors. These varied sensors offer remarkable potential and are used to measure temperature, stress, concentration, the presence of gas, vibrations, magnetic fields, and humidity.

In recent years, breakthroughs in nanofiber techniques have led to the successful combination of nanofibers and commercialized products and created a new wave of nanofiber-based inventions [6,7]. Electrospinning is the simplest method of producing nanofibers; the burgeoning use of nanofibers drives the development of new electrospinning techniques. Electrospinning uses polymers to produce nanofiber films, various variety of commercial products based on nanofibers that have extensive use in our everyday life, such as membranes nanofibers in biomedical scaffolds [8], electrospun nanofibers in energy storage components [9]. Millimeter- or nanometer-scale fibers are prepared by exposing polymer solutions to high-voltage electrostatic forces. The materials required for this process are widely available, and the process is relatively simple, compared to conventional spinning techniques. The complex compound approach in material design allows developing nanosystem and obtaining a segregated 3D network of nanomaterial through formula types of nanofiber, nanotube, nanocluster, which can significant enhancement of mechanical, chemical, electrical, and optical properties [10,11].

Moreover, electrospun products have better properties such as smaller fiber diameters, higher specific surface areas, and higher porosities. During the spinning process, fiber structures are affected by various factors, including the properties of the materials, the concentration of the polymer solution, the discharge voltage, and the distance between the nozzle and the collection plate. Studies across many disciplines have examined special fiber structures, such as composite and porous, to expand the range of possible applications [12,13]. Electrospun products used in biomedicine are mostly fabricated from natural polymers [14]. Therefore, electrospun products are widely applied and the vast potential of electrospinning has yet to be fully exploited. GQDs are small graphene fragments in which electron transport is limited in all three spatial dimensions [15]. The valence band and conduction band of GQDs overlap slightly, which makes this material a semiconductor. The excitons in graphene have an infinite Bohr diameter. Therefore, graphene fragments of any size will exhibit quantum confinement effects. Graphene absorbs a relatively high proportion of light and hardly depends on the wavelength in its visible spectrum. This indicates that the absorption of graphene is saturated above a certain intensity of incident light [16]. In addition, it has been observed that the refractive index of graphene changes drastically when an electric field is applied [17,18]. The main difference between GQD and semiconductor QD photoluminescence (PL) is that the PL bandwidth of GQD is much wider [19].

In 1910, the English physicist Lord Rayleigh introduced the whispering gallery wave phenomenon, also known as whispering gallery modes (WGM) [20]. This phenomenon explains how sound waves travel similarly to light traveling in optical fibers, that is, light waves travel on the surface of optical fibers after experiencing total reflection. Optical sensors coated with electrospun nanofibrous membranes were first introduced by Xianyan Wang et al. [21] in 2002. The optical sensors were electrospun using a solution of poly(acrylic acid)−poly(pyrene methanol) (PAA−PM) and thermally cross-linkable polyurethane–latex. These sensors exhibited higher sensitivities because of the high specific surface areas of the nanofibrous membrane structures. In 2016, Petronela Pascariu et al. [22] fabricated NiO–SnO_2_ ceramic nanofibers via electrospinning. Electrical measurements conducted over a relative humidity (RH) range from 0% to 100% tested the humidity-sensing properties of the nanofibers. The results showed that the electrical resistivity of the sensors gradually decreased, thus proving that NiO–SnO_2_ nanofibers can be used as the active nanostructures in humidity sensors. In 2015, Kyung Hyun Choi et al. [23] used composite polyvinyl alcohol (PVA)–GQDs sensing layers to prepare a high-performance humidity sensor for environmental and health monitoring. The sensing layer was first deposited on an interdigitated transducer (IDT) printed on a piezoelectric substrate. Afterward, the electrical properties of the sensor were determined in order to examine the response at different humidity levels. The results indicated that the sensor had excellent responses, specifically, a response time of 0.625 s and a recovery time of 0.53 s. Therefore, the sensor responded well to increasing and decreasing humidity levels. In 2018, Ce Bian et al. [24] proposed an optical fiber humidity sensor based on an optical Fabry–Perot interferometer. The sensor was made by coating a polyimide (PI) film onto a short section of a hollow-core fiber. Experiments were performed by exploiting the direct responses of the PI film. The results revealed that the sensor attained a sensitivity of up to 1.309 nm/%RH within 40% RH–80% RH. In 2018, Rodrigues, Bruno VM et al. [25] proposed a simple, low-cost, and fast method that uses electrospinning technology to make PVA/water-soluble GQDs into a fluorescent fiber. In 2019, Guo [26] developed a bending-type biconical humidity sensor using a fused biconical taper and heat-setting processes. A fluorescent moisture-sensitive film was coated on the sensor to enhance its moisture-sensing performance. In addition, the authors investigated sensors with different structures and found that smaller radii of curvature and conical diameters, as well as longer biconical lengths, can increase the sensitivity and linearity of the sensor. In 2019, Zhao et al. [27] constructed a U-shaped probe-type humidity sensor and improved its sensitivity by coating it with a layer of PVA film. This sensor had a wavelength sensitivity of 318.1 pm/%RH within a range of 15% to 85% RH.

Combining and building on these previous studies, this research employed electrospinning techniques to coat a layer of composite PVA–GQDs film onto the surface of a U-shaped optical fiber probe sensor for humidity measurement applications. The high specific surface area, as well as the specificity of the electrospun film of the material, could improve the sensitivity of the sensor during the humidity measurements.

## 2. Theory

### 2.1. The Wavelength–Bend Radius Relation

In 1992, Hagen Renner et al. [28] proposed that when single-mode optical fibers experience bending, the transverse field distribution *ψ*(*x*,*y*) in the optical waveguides can be expressed as the two-dimensional scalar equation, as shown in Equation (1).

(1) $$\nabla_{t}^{2}\psi \left(x,y\right)+\left[k^{2}n_{eff}^{2}\left(x,y\right)-\beta^{2}\right]\psi \left(x,y\right)=0$$

where k=2π/λ, and β and λ represent, respectively, the propagation constant and the wavelength of the basic leakage mode.$\;n_{2}$ and $n_{3}$ are the refractive indices of the cladding and the surface coating of the optical fiber, while a and b represent the radii of the core and the cladding, respectively. When x≥a, the effective refractive index distribution in the bent fiber can be expressed as Equation (2).

(2) $$n_{eff}^{2}\left(x,y\right)=n^{2}\left(x,y\right)\left(1+\frac{2x}{R}\right)$$

where $n^{2}\left(x,y\right)\;$represents the refractive index of the unbentoptical fiber. The effective refractive index $n_{eff}\left(x,y\right)\;$changes according to the diameter (x) and the bend radius (*R*) of the optical fiber. Since optical fibers of various diameters were used in this study, Equation (3) can be deduced by revising and rearranging Equation (2) as follows:

(3) $$\lambda \times {\left(\Delta \lambda \right)}^{-\frac{1}{2}}={\left[\frac{4n_{eff}^{2}}{3}\sqrt{\frac{2b^{3}}{R}}\right]}^{\frac{1}{2}},\;R\ll R_{C}$$

From Equation (3), when $R\ll R_{C}$, the ratio of the wavelength position can be derived from the bend radius (R), the radius of the cladding (b), and the effective refractive index ($n_{eff}$). According to the previous wavelength-related equations, the wavelength shifts are primarily affected by three parameters—the diameter of the optical fiber, the bend radius, and the effective refractive index.

### 2.2. The Wavelength–Refractive Index Relation

This section discusses the sensitivity of the refractive index to the wavelength. According to Lu et al. [29] in 2014 and Zhang et al. [30] in 2015, a leakage mode forms in the bending region of an optical fiber with a bend radius small enough that when light enters the bending region, total reflection is no longer maintained at the interface between the core and cladding layers. Thus, some light rays can leak or be emitted from the core layer to the cladding layer and reflect at the interface between the external media and the cladding layer, forming a WGM. The light rays would then be coupled back to the core layer. When the light rays couple via the WGM from the cladding layer to the core layer, interference is formed due to the two different transmission pathways. As a result, a special WGM wavelength spectrum is generated, in which multiple peaks and troughs can be observed.

The interference spectrum light source intensity of the WGM can be represented by Equation (4) [28].

(4) $$I=I_{co}+I_{wis}+2\sqrt{I_{co}+I_{wis}}\cos\left(\phi \right)$$

where $I_{co}$ and $I_{wis}\;$are the intensity of light in the core mode of the fiber and the WGM, respectively; ϕ is the phase difference between the core mode and the WGM. It is known that an increase in the refractive index would alter the effective refractive index difference of the core and cladding, and the position of the wavelength at the point of loss would change according to the refractive index. The wavelength sensitivity can be expressed as Equation (5) based on the refractive index variations in the external environment.

(5) $$\frac{d\lambda_{D}}{dn_{ext}}=\frac{-\lambda_{D}}{\Delta n_{eff}}\frac{\partial n_{eff}^{cl,m}}{\partial n_{ext}}/\left[1-\frac{\lambda_{D}}{\Delta n_{eff}}\left(\frac{\partial n_{eff}^{co}}{\partial \lambda }-\frac{\partial n_{eff}^{cl,m}}{\partial \lambda }\right)\right]$$

The equations relating the refractive index and the sensitivity as proposed by the scholars above show that the effective refractive indices of the core and cladding modes should vary when the external refractive index changes, which would cause wavelength shifts. The effective refractive index of the core of a typical single-mode optical fiber is greater than that of its cladding, and its effective refractive index equation can be written as $n_{eff}^{co}-n_{eff}^{cl,m}>0$.

In addition, when $\frac{\lambda_{D}}{\Delta n_{eff}}\left(\frac{\partial n_{eff}^{co}}{\partial \lambda }-\frac{\partial n_{eff}^{cl,m}}{\partial \lambda }\right)<1$, the wavelength sensitivity to refractive index variations becomes negative, which means that any increase in the refractive index would decrease the wavelength.

## 3. Fabrication Process and Experimental Setup

### 3.1. Fabrication of the U-Shaped Optical Fiber Probe Sensor

A 3 cm section was stripped from the center of a single-mode optical fiber (Corning SMF28) to prepare the U-shaped optical fiber probe sensor. The diameter of the core and cladding was 125 µm and 10 µm. The SMF28 fiber consists of all glass and supports single-mode light propagation at a 1310/1550 nm operating wavelength and the effective index of refraction was 1.4682 at a 1550 nm operating wavelength. Next, the single-mode optical fiber was fabricated into a U shape using a flame heating process. The completed U-shaped structure was removed and packaged inside a quartz glass tube. Electrospinning was employed to coat both sides of the U-shaped optical fiber probe sensor with thread structures, completing the probe sensor.

### 3.2. Flame Heating Method for Fabricating Optical Fibers

**Figure 1:**
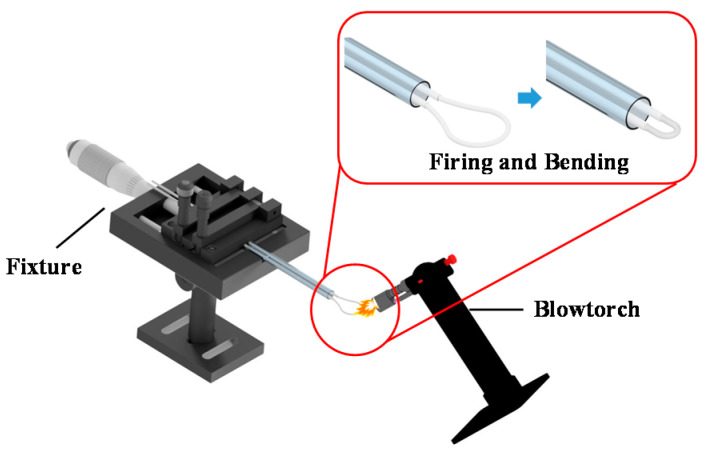
Illustration of the fabrication of a U-shaped optical fiber via a 3 cm bare single-mode optical fiber was bent into a wide loop and placed into a mold for flame heating. The fixture adjusts simultaneously for setup to reduce the loop at the same time.

First, a 3 cm section of the protective layer in the middle of the single-mode optical fiber was removed. Next, the two ends of the optical fiber were put into a hollow glass tube and fixed in the glass tube with UV resin; the fixture was used to control the diameter of the U-shaped fiber probe. As shown in Figure 1, the exposed fiber was bent into a wide loop and placed into a mold for heating. The mold was secured onto a stage, and flame from a gas blowtorch was directed onto the loop. When the temperature was sufficiently high, the bend radius of the optical fiber was reduced to the point that the loop became a U shape that fit into the preset diameter of the mold.

### 3.3. Fabrication of the Quartz Glass Tube Package

**Figure 2:**
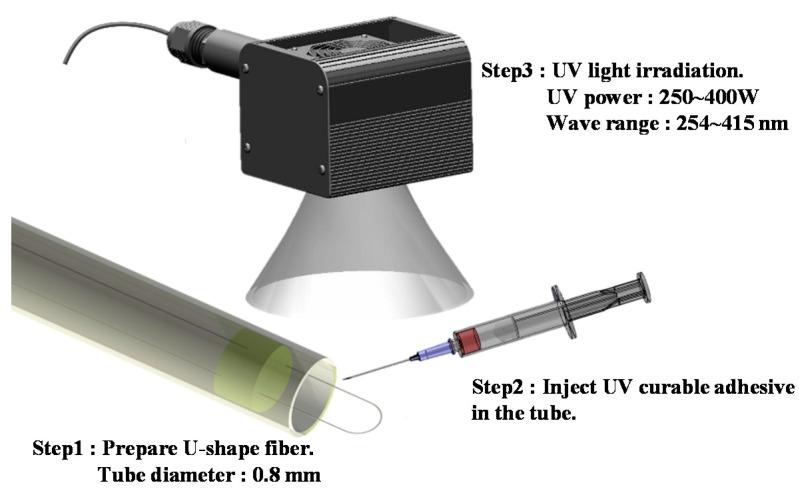
Schematic of the quartz glass tube packaging process. First through UV curable adhesive and glass tube fixed single-mode optical fiber was bent into a wide loop. Then, to ensure the diameter remains unchanged by UV adhesive under UV light was exposed.

Since the U-shaped optical fiber probe sensor is extremely sensitive to changes in its diameter, its packaging must be adequate to ensure that the diameter remains unchanged and the measurements are unaffected by external factors. First, the U-shaped optical fiber was placed into a quartz glass tube and slightly adjusted and both ends of fiber were fixed on a micro platform as step 1. The diameter of the hole depends on the final bending radius. The metal orifice slat itself was fixed on stage number. A UV curing adhesive was pumped from a syringe onto the front end of the quartz glass tube as step 2, and the packaging was cured by irradiation with UV light as step 3. The U-shaped optical fiber probe sensor was ready for use after the package had cooled down, as shown in Figure 2.

### 3.4. PVA/GQDs Mixed Solution Preparation

The preparation steps of the PVA and PVA/GQDs mixed solution were as follows: Firstly, 1.2 g of PVA (MW 89,000–98,000, Sigma-Aldrich Chemie GmbH, Eschenstrasse 5 D-82024 TAUFKIRCHEN, Germany) was added to 10 mL of deionized water. Then, the solution was stirred at 80 °C for one hour by the magnetic stirrer until it was completely distributed dissolved and evenly transparent. Then, 12 wt.% PVA solution was mixed with GQDs (0.008 wt.%) dispersion (GQDs, Sigma-Aldrich with a concentration of 1 mg/mL in H_2_O and a particle size of 5 nm). GQDs had emitting wavelength at 445 nm, quantum yield >65%. Lastly, the PVA/GQDs mixed solution was mixed by the magnetic stirrer oscillated for 24 h at room temperature to make them uniform.

### 3.5. Preparation of the Electrospun Film

The spinning polymer solution of 12.0 wt.% PVA and 12.0 wt.% PVA doped with water-soluble graphene quantum dots were prepared separately. The U-shaped optical fiber probe sensor was secured onto a collection plate covered with copper tape. A syringe was secured on a plastic clamp and positioned so that the tips of the needle and the collection plate were 15 cm apart. An infusion pump operating at a constant speed pumped polymer solution into the syringe through a capillary tube. After ensuring that no air pockets were present inside the tube, and the polymer solution was supplied to the needle without interruption, 17,000 V were applied to the syringe while the copper tape on the collection plate was connected to a ground wire. Charged threads formed between the needle and the plate, and these were uniformly coated onto the surface of the U-shaped optical fiber for 10 min. The same process was performed on the other side of the fiber. Therefore, both sides of the U-shaped optical fiber were uniformly coated with electrospun thread structures, and the completed optical fiber sensor was removed with the aid of tools. The environmental temperature and humidity were controlled at 25 °C and 55% RH.

### 3.6. Experimental Setup for Humidity Measurements

**Figure 3:**
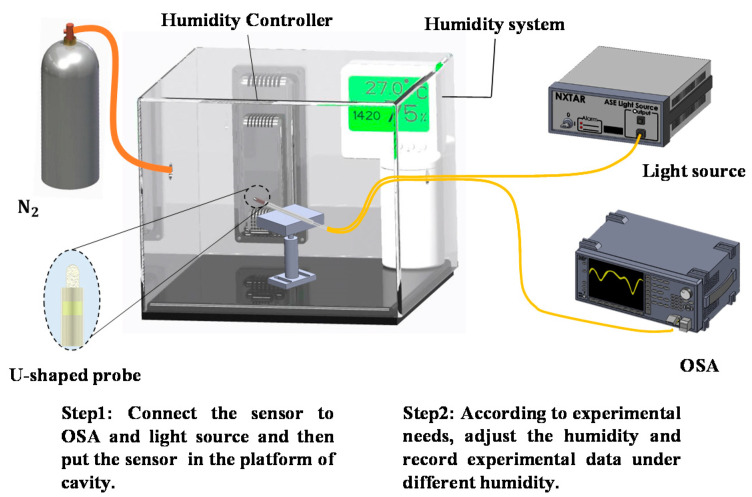
Schematic of the experimental setup for humidity measurements. A U-shaped sensor was secured onto a stage inside the control box. the sensor was connected to the light source and OSA. Humidifier was used to increasing humidity and nitrogen gas was used to reduce the humidity.

The sensors were placed in a chamber that contains an ultrasonic nebulizer that increases the humidity, which is countered by nitrogen gas flow to decrease the humidity, and a humidity monitoring system. As shown in Figure 3, the electrospun-coated U-shaped optical fiber probe sensor was secured onto a stage inside the control box of a humidity simulation system. Each step of fabrication and detection was performed in the optical table and platform of cavity systems (M-TC120X-2A, OP Mount Instrument Inc., Pingzhen Dist, Taoyuan City, 324 Taiwan) to prevent vibration. One end of the optical fiber sensor was connected to a broadband light source, while the other end was connected to an OSA. To achieve a humidity level of 80% RH inside the control box, water vapor was produced using a water mist system; nitrogen gas was used to reduce the humidity level to 20% RH. The variation in the humidity level throughout the entire experimental process was monitored using a humidity indicator. The variations in the observed wavelength and transmission loss were monitored using the OSA (MS9710C, Anritsu, Taipei, 114, Taiwan). The experiments were performed over three cycles, and the data were collated.

## 4. Results and Discussion

That concentration of 8, 10, 12, and 14 wt.% PVA (Mw 89,000–98,000) polymer solutions were prepared separately. Polymer fiber’s shape could not throughput continuously via formula applied used 8 and 10 wt.% PVA polymer solutions in the electrospun process. Due to the high viscosity of 14 wt.% PVA polymer solutions, fiber diameter becomes larger and performed using a larger high voltage generator with a direct current (DC). The positive electrode was connected to a metal needle that a high voltage electric field was formed between the positive and negative electrodes to create the electrospun region, while the 12 wt.% PVA solution was conducted fiber shape continuously. Therefore, nanofiber through 12 wt.% PVA solution was formed.

**Figure 4:**
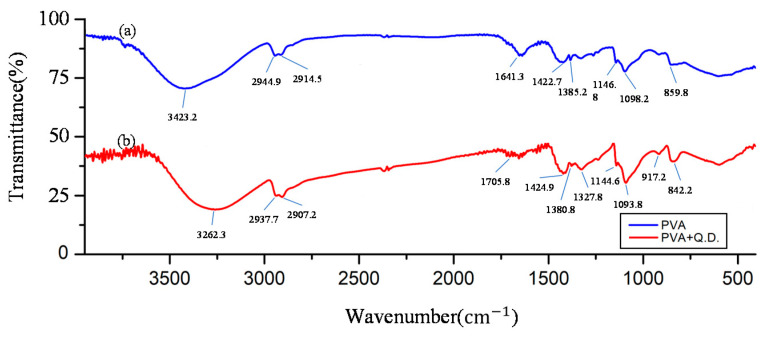
FTIR spectra in nanofiber of (**a**) PVA; (**b**) GQDs are doped into PVA fabricated via electrospun technology.

The interactions between the PVA and GQDs were indicated by Fourier transform infrared (FTIR) spectroscopy. As shown in Figure 4, a comparison between the FTIR spectra of PVA (Figure 4a) and GQDs doped in PVA (Figure 4b). The FTIR spectroscopy of PVA showed bands at 3423.2, 2944.9, 2914.5, 1641.3, 1422.7, 1385.2, 1146.8, 1098.2, 859.8 cm^−1^ and the FTIR spectroscopy of PVA/GQDs showed bands at 3262.3, 2937.7, 2907.2, 1705.8, 1424.9, 1380.8, 1144.6, 1093.8, 842.2 cm^−1^. They related to the vibrations of -OH, -CH_2_ asymmetric, -CH_2_ symmetric stretching, C=O stretching in acetate group, CH_2_ wagging, -CH_2_ stretching, C-H wagging, C-O stretching of the acetyl group, and C-C [31]. Figure 4 depicts the FTIR spectrum of the GQDs doped with PVA nanofiber, the band of –OH asymmetric at 3262.3 cm^−1^, and C-O stretching at and 1093.8 cm^−1^ [32,33], implying the vibration of hydroxyl group and carboxyl group that functionalized onto crystalline GQDs. FTIR spectroscopy is ultra-sensitive to the formation of hydrogen bonds. The H-bond formed causes a peak of O-H stretching vibration to disappear at 3262.3 cm^−1^ after doped GQDs in PVA nanofiber. As regards the FTIR spectra of doped GQDs in PVA polymer fiber, they present changes in the intensities of some bands and shifts in other bands, compared with undoped PVA especially in hydrogen bond and C-O bond.

In this study, electrospinning techniques were applied to fabricate U-shaped optical fiber probe sensors using a PVA and a composite PVA–GQDs solution. Both sides of the sensor were uniformly coated with electrospun films, and the finished product was placed into a humidity simulation control box for test measurements. The measurements were conducted at humidity levels ranging from 20% RH to 80% RH, and measurements were recorded at 10% RH intervals. That the transmission response and resonance wavelength shift induced by exposure to H_2_O gas over three cycles with the same optical sensor. The humidity levels were first increased and then decreased, and the resulting wavelength shifts were analyzed.

**Figure 5:**
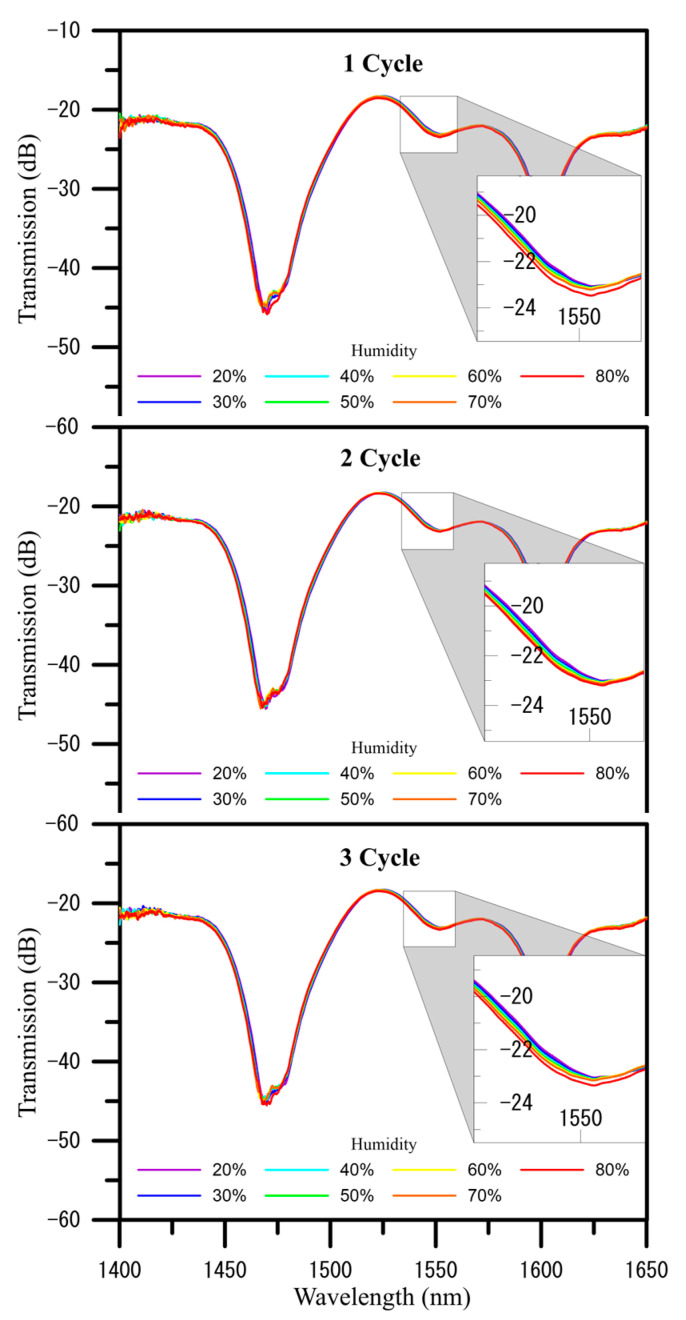
Spectra of transmission loss and resonance wavelength shifts induced over three cycles in a U-shaped optical fiber sensor coated with electrospun PVA nanofibers when humidity was increased from 20% to 80% RH.

**Figure 6:**
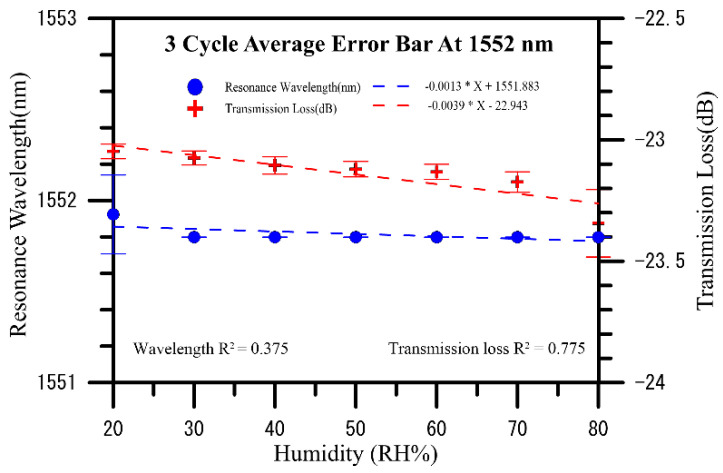
Analyses of transmission loss and resonance wavelength shifts induced over three cycles in the U-shaped optical fiber sensor coated with electrospun PVA nanofibers when humidity was increased from 20% to 80% RH.

Figure 5 shows the humidity measurements of a PVA nanofiber coated U-shaped optical fiber sensor over three cycles after increasing the humidity level from 20% RH to 80%RH blueshifts the initial wavelength of 1552.174 nm to a wavelength of 1551.799 nm, for a total shift of 0.375 nm, and their analysis chart of wavelength and transmission loss of humidity measurements performed are shown in Figure 6. The three-cycle spectrogram can be found that as the RH% humidity increases, the dip value in the spectrum will decrease the wavelength, indicating that the wavelength shifts to blue wavelengths and the overall loss (transmission) become smaller slightly. In three cycles of humidity measurement, the average sensitivity of transmission to humidity is −0.0039 dB/%RH, and the average sensitivity of wavelength to humidity is −0.0013 nm/%RH.

**Figure 7:**
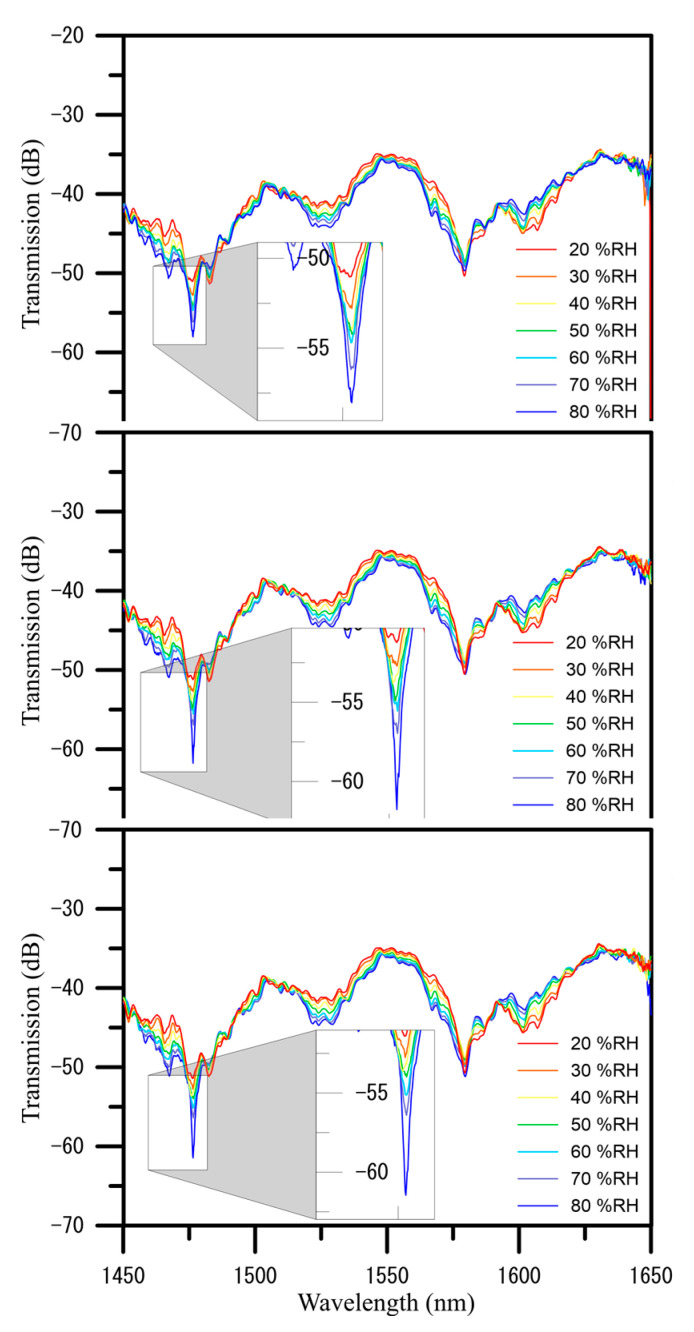
Spectra of transmission loss and resonance wavelength shifts induced over three cycles in a U-shaped optical fiber sensor coated with electrospun graphene quantum dots doped PVA nanofibers GQDUS when humidity was increased from 20% to 80% RH.

**Figure 8:**
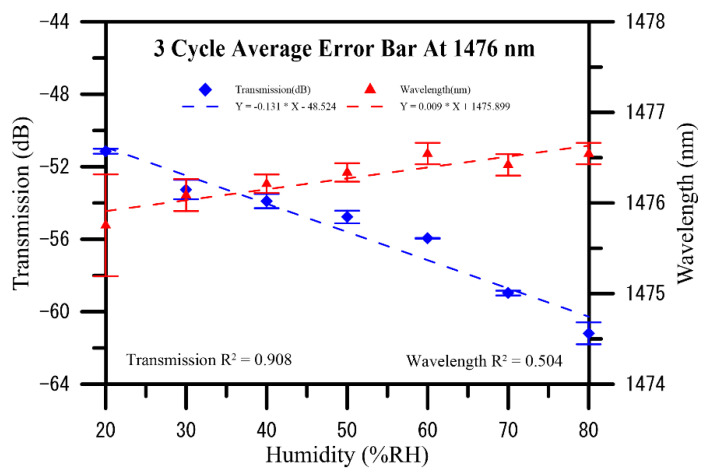
Analyses of transmission loss and resonance wavelength shifts induced over three cycles in the U-shaped optical fiber sensor coated with electrospun graphene quantum dots doped PVA nanofibers GQDUS when humidity was increased from 20% to 80% RH.

During the U-shaped optical fiber probe sensors coated using a composite PVA–GQDs nanofiber experimental, increasing humidity resulted in gradual enhancement in variations in the wavelength shift, the spectra and analyses performed in error bars of humidity measurements in three cycles, as shown in Figure 7; Figure 8, respectively. The humidity level from 20% RH to 80% RH redshifts the initial wavelength in three cycles of humidity measurement, the average sensitivity of transmission to humidity is –0.131 dB/%RH, and the average sensitivity of wavelength to humidity is 0.009 nm/%RH. More specifically, the wavelength changed to redshift as humidity increased. The effects of each type of sensing layer were compared when the humidity varied in Table 1. The resonance wavelength of PVA–GQDs nanofiber sensors will redshift since the slight PL increases nanofiber-refracted index changes of the sensor when GQDs are used as the doped sensing layer. Only a tiny shift is observed in the resonant wavelength when the independent PVA nanofiber component is the sensing layer. This phenomenon is attributed, according to Equation (5), to the fact that the resonance wavelength of the core mode is affected by environmental RI causing GQDs PL. Density functional theory (DFT) calculations show that in GQDs composed of 20 aromatic rings, the bandgap of GQDs increases to approximately 2 eV, while that of benzene is 7 eV.

The absorption spectrum of GQDs shows a peak at approximately 230 nm, which is designated as the π → π* transitions of the aromatic sp^2^ domains in the graphene lattice. The dip of sensors spectrum and their maximum shifts to red and decreases with the increase of humidity. Although the phenomenon has been clearly observed, the actual mechanism of sensitivity increasing in GQDs is still unclear. It has been suggested that the photoluminescence emission occurs when the electrons relax to the σ-orbital, and a shortened emission wavelength is observed. We propose that is what causes the increased sensitivity [34].

**Figure 9:**
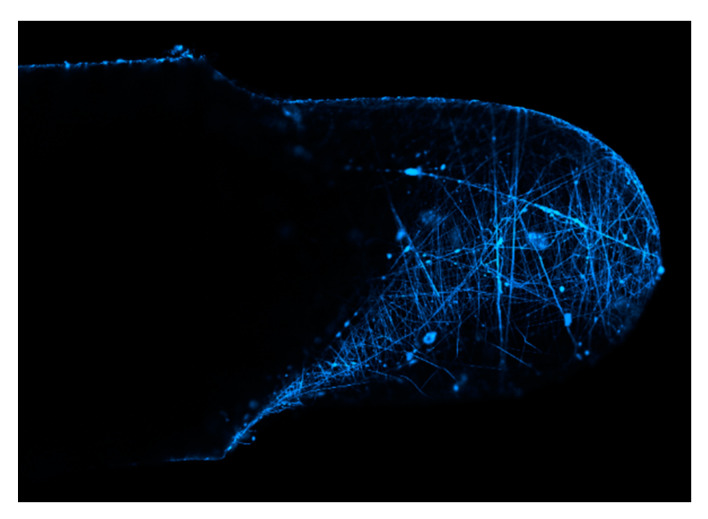
Confocal images of GQDUS of fluorescence excita.

Electron microscope confocal images of PVA/GQDs nanofiber coated U-shaped fiber sensor obtained (Figure 9) were processed by using NIKON A1. Confocal image luminescence intensity profile representative photos showed significant potential fluorescence luminesces. The experimental results above indicate that the U-shaped optical fiber probe sensor with both sides uniformly coated with composite PVA/GQDs demonstrated a wavelength shift sensitivity increasing from 0.0013 to 0.0090 nm/% RH compare with PVA independent nanofiber during the humidity measurements. The U-shaped optical fiber probe sensor coated with a PVA/GQDs nanofiber composite polymer film displayed excellent performance in humidity measurements. In three cycles of humidity measurement, the average sensitivity of transmission to humidity is 0.131 dB/%RH, and the average sensitivity of wavelength to humidity is 0.009 nm/%RH. It appears these two types of nanofibers can play an important role in the development of the proposed sensor. The electrospinning results in a composite of PVA/GQDs nanofiber in higher sensitivity than PVA both in transmission and wavelength. The present study thus proposes a convenient technique that can be performed at room temperature for humidity detection using polymer nanofiber. The feature analysis of quantum dots doped polymer will be further analyzed with more sensitive sensors’ sensitive responses. This result will provide further development and utilization in the sensor field.

## 5. Conclusions

A graphene quantum dots sensor was successfully fabricated and to enhance sensitivity, coated with a composite solution consisting of GQDs doped polymer on both sides via electrospinning, and used for humidity measurements. Over a range of 20% RH to 80% RH, the sensor had a wavelength shift sensitivity of 0.009 nm/%RH, and sensitivity of transmission is 0.131 dB/%RH, indicating that the electrospun U-shaped optical fiber probe sensor has great potential in research and development. Through confocal image luminescence intensity profile could be noticed obviously. In the future, different materials could be electrospun onto the U-shaped optical fiber probe sensor to test the suitability of the materials for measuring different physical quantities, such as temperature, humidity, and concentration. Natural polymers could also potentially be used to coat the U-shaped optical fiber probe for biomedical measurements. The optical fiber humidity sensor in this experiment was successfully mixed with PVA with quantum dots, and electrospun to make a nanofiber mesh and coat it on the U-shaped optical fiber for the humidity sensing experiment. It can be seen from the experimental results that the average wavelength drift is about 0.009 nm/%RH, and the average loss change is about 0.131 dB/%RH. This U-shaped optical fiber humidity sensor has enhanced wavelength and loss response sensitivity to humidity.

## Figures and Tables

**Table 1:** Average sensitivity of wavelength and transmission consisted through U-shaped optical fiber probe sensors coated using a PVA and a composite PVA–GQDs nanofiber.

Sensing Material	Wavelength (nm/%RH)	Transmission (dB/%RH)
Nano fiber PVA	−0.0013	−0.0039
Nano fiber PVA/GQDs	0.009	−0.131

## Data Availability

Not applicable.
